# Refuting ‘a new theory for X-ray diffraction’ – a reciprocal-space approach

**DOI:** 10.1107/S2053273325000762

**Published:** 2025-05-27

**Authors:** Elias Vlieg, Paul Tinnemans, René de Gelder

**Affiliations:** a Radboud University, Institute for Molecules and Materials, Heyendaalseweg 135, Nijmegen, 6525AJ, The Netherlands; Institute of Crystallography - CNR, Bari, Italy

**Keywords:** X-ray diffraction, scattering theory, crystal termination

## Abstract

Some ten years ago, Fewster proposed ‘a new theory for X-ray diffraction’ in order to explain the completeness of powder diffraction patterns from samples with very few crystals, claiming to find extra intensity at Bragg scattering angles 2θ_B_, even when a grain was not oriented in the Bragg condition, and claiming this to be a new approach to X-ray scattering [Fewster (2014). *Acta Cryst.* A**70**, 257–282]. Fraser & Wark [*Acta Cryst.* (2018), A**74**, 447–456] gave a detailed account of the errors and issues in the approach by Fewster, but the situation appears to be still undecided. To address this issue, we use a different perspective, based on conventional scattering theory and using a simpler description in reciprocal space, rather than the angular space used by Fewster and by Fraser & Wark. This allows us to focus on the crucial conceptual errors in the proposed theory. We show that Fewster is in fact not proposing a new theory, but finds effects that disagree with conventional theory because of errors in the path length calculation. We also discuss extensively the effect of residual intensity in reciprocal space, away from the Bragg peaks, and caused by the termination of crystals. We show that the residual intensity has no significant effect on the intensity of typical powder diffraction patterns. We hope that, with this account, we can put the discussion about the new theory to rest, along with the theory itself.

## Abstract 2

A ‘new theory for X-ray diffraction’ proposed by Fewster in 2014 is shown to be based on a conceptual error. Residual intensity away from Bragg peaks is also discussed.

## 1. Introduction

In 2014 Fewster published a paper with the title ‘A new theory for X-ray diffraction’ (Fewster, 2014 ▸) (called F14 from now on). The motivation for developing this new theory was the observation that the diffraction from only a small number of crystals can already yield complete powder diffraction patterns. We will present our results on powder diffraction from small samples in a future publication, but here we want to address the ideas put forward in F14. Fraser & Wark (2018 ▸) wrote a detailed critique (called FW18 from now on) of the new theory, showing that the claims from F14 are in error. We largely agree with the arguments in FW18 and do not aim to repeat this account. Fewster himself did not agree with FW18, as he expressed in his response (Fewster, 2018*a* ▸) (that we will call F18 from now on) and in a number of subsequent publications (Fewster, 2018*b* ▸; Fewster, 2023 ▸). The amount of mathematics and the use of angle parameters in these references may obscure the main arguments, and so part of the X-ray diffraction community may be under the impression that the issue is unresolved. We use a description based on the physics of X-ray scattering and on reciprocal space instead of angular space. The aim of this article is therefore to give as short an account as possible and focus on the key misconceptions and errors; in this way we demonstrate that the new theory is indeed wrong. We are also in a position to address the arguments in F18 and do this where relevant. F14 has, nevertheless, raised a number of interesting points, in particular concerning the presence and distribution of additional intensity in reciprocal space. This is well known in the conventional theory, and we will show that this residual intensity has no significant effect on the measured intensity of a Bragg peak.

## 2. The main errors in the new theory

In the *Introduction* of F14, it is stated that ‘An alternative viewpoint is presented here, where the whole of diffraction space is occupied by scattering from many crystal planes, which when combined contribute to the peaks observed’. We agree with this description of diffraction, but stress that this is standard X-ray scattering theory and not an alternative viewpoint. Nevertheless, using this same basic methodology, F14 should have arrived at the same result as the conventional theory if applied correctly. We will first show that the amplitude derived in F14 is wrong, then point out the mistakes in the path length calculation that lead to the wrong amplitude, and finally emphasize the major misconception that resulted from this and that formed the basis of the new theory.

### 2.1. The scattering amplitude

**Figure 1:**
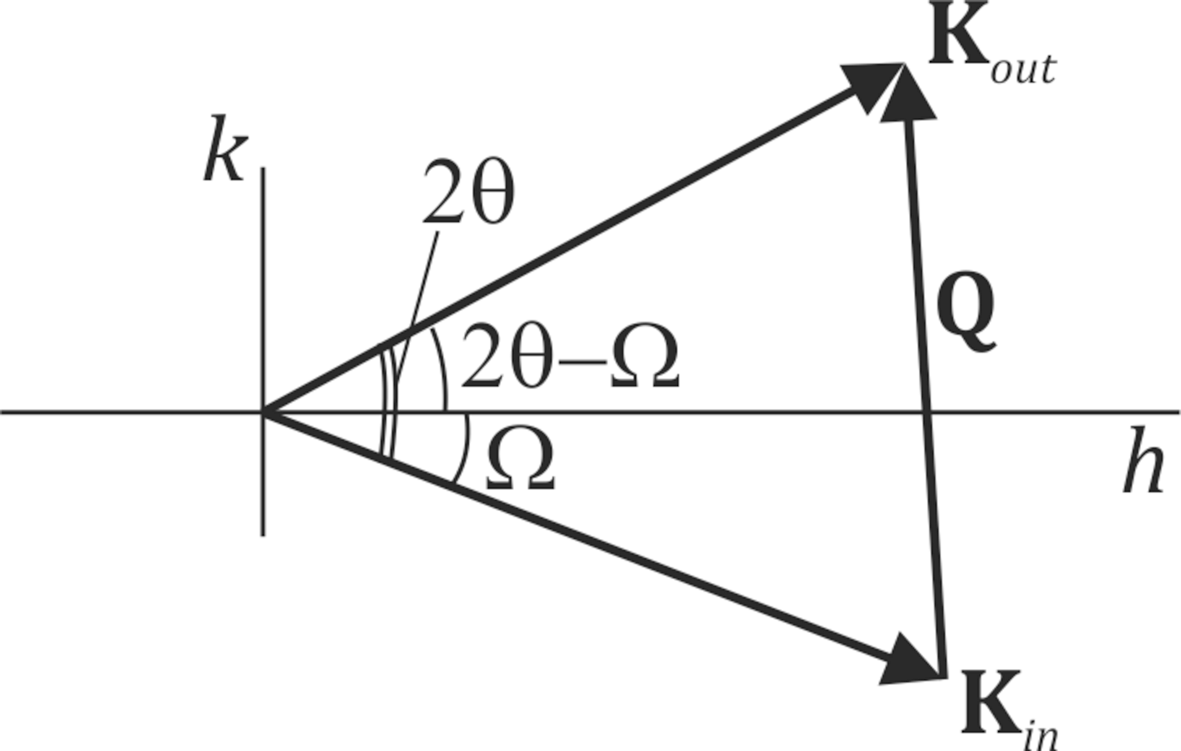
The incoming and outgoing wavevectors, oriented such that the total scattering angle is 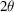. The incoming angle is Ω.

In F14 a 2D model for a crystal is used, in which the lattice planes are represented as planes (lines to be precise) of uniform electron density. The conventional theory yields for this case the following expression for the scattering amplitude: 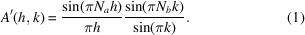
Here *h* and *k* are the diffraction indices that can have *real* (not only integer) values and 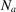 and 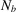 are the number of unit cells along the crystallographic *a* and *b* directions. Section *A*1 of the Appendix[App appa] gives a summary of conventional theory, and we refer to this for details on the derivation of the scattering amplitude. F14 does not use diffraction indices to represent a specific scattering geometry, but incoming and outgoing angles with respect to the scattering planes, and therefore we will need to convert equation (1) to the angular coordinates used in F14. Fig. 1 ▸ shows the geometry and scattering angles. It is straightforward to show that the following relation holds: 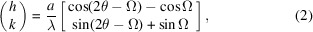
where *a* is the lattice constant, λ the wavelength, Ω the incoming angle and 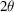 the scattering angle. We refer to Section *A*2 of the Appendix for details. Substituting the full expressions for *h* and *k* in equation (1), we obtain
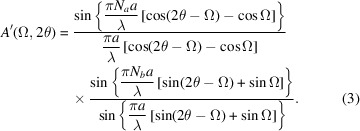
This expression is, as it should be, the same as that presented by FW18 as equation (5), except that they did not use the diffraction plane approximation and thus have a sine term in the left-most denominator. Both equation (1), using diffraction indices, and equation (3), using angular coordinates, are based on conventional theory and cover all possible scattering geometries. F18 thus clearly makes a mistake by stating ‘What happens if the detector is moved to a different 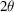 angle, whilst maintaining the same incident angle? The description of Fraser & Wark or the conventional theory does not consider this’. This is not true, the conventional theory *does* consider this.

Now we compare our result with equation (F14-5). The first part of equation (F14-5) is the same as equation (F14-4), which we copy here for convenience: 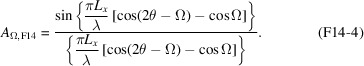
Note that 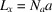. It is nearly the same as the first term in equation (3), except that the denominator contains an 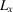 term that should not be there. This gives a much lower amplitude, but otherwise does not affect the behaviour as a function of the angles 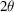 and Ω. Equation (F14-4) was not derived in F14, so it is unclear why the 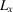 term is present, but it might be a simple typing error. Except for this detail, the equations agree and this shows that the conversion from diffraction indices to angles works properly. The significant differences, however, are found in the second term, describing the interference between planes. We copy this part of equation (F14-5) for convenience as well: 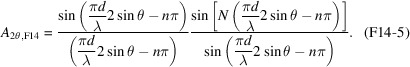
For the comparison, we should use 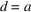 and 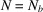. The *n* in this equation corresponds to a path length difference of multiple wavelengths. While there are similarities, the differences are profound and the two terms clearly do not agree with each other. We claim the expression we use is the correct one because (i) it is based on a standard result that can be found in several textbooks; (ii) it is essential in the computation to add all the contributions with the correct phase and this is guaranteed in our case by using the standard expressions for path length difference. In F14, however, the phases are calculated in an elaborate, but incorrect way, as explained by FW18 and in our next section. It is unfortunate that F18 does not contain a response to the mathematical errors and arguments as pointed out by FW18, nor provide more details on how equation (F14-5) was derived. We conclude, as did FW18 before us, that the scattering amplitude used in F14 is wrong.

### 2.2. Path length difference

**Figure 2:**
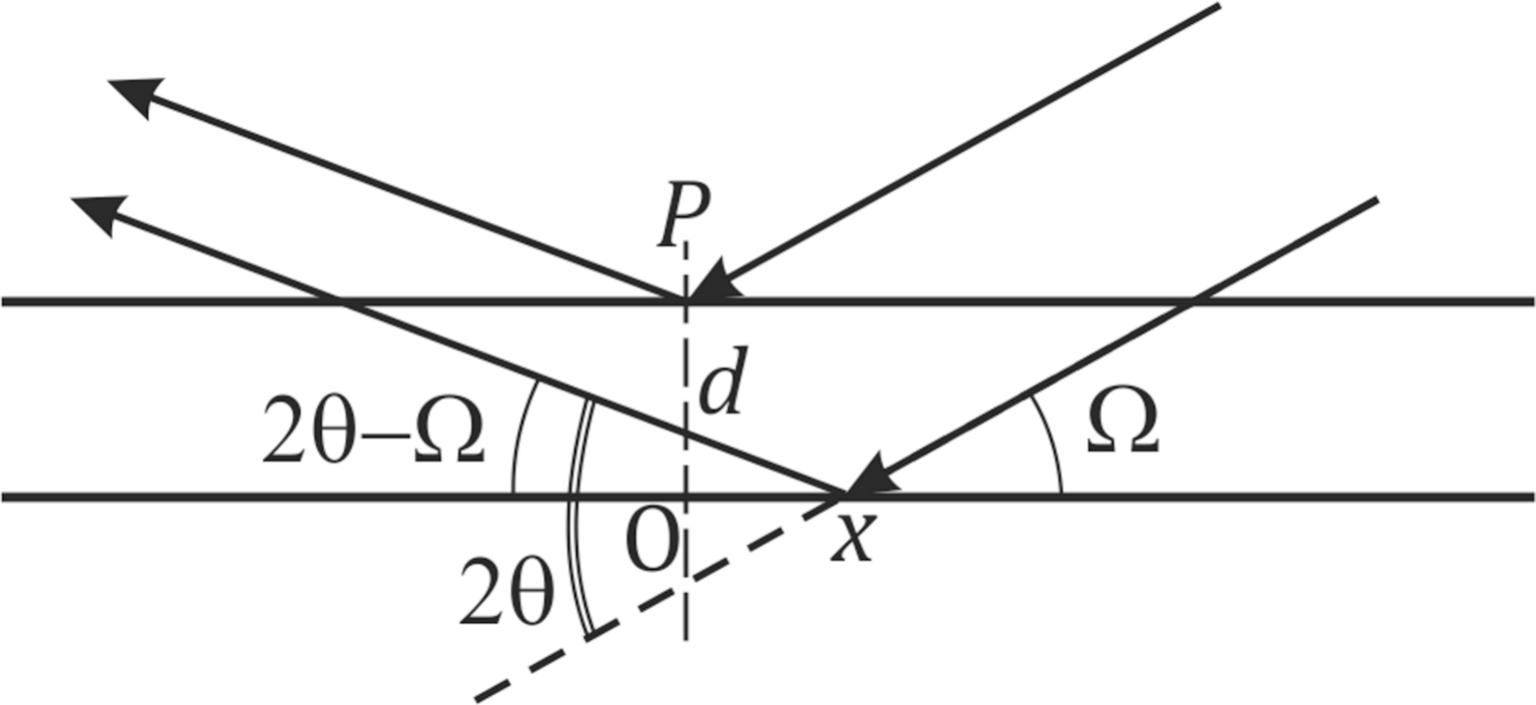
The scattering geometry for computing the path length difference between point *P* on the top plane and a point *x* on the lower plane. O is the origin on the lower plane. This is an adaptation of Fig. 4(*d*) from F14.

At the Bragg condition, where the scattering angle has the value 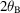 and 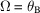, the scattering amplitudes from all parallel planes are in phase. (This is true for a series of *n* angles, following Bragg’s law 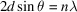, but we will use 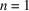 as ‘the’ Bragg angle.) In order to demonstrate that two parallel planes can be in phase when 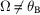, F14 computes the path length difference for a point on the top plane and a point on the next lower plane that is laterally displaced by a distance *x*. Fig. 2 ▸ shows the geometry used. In F14 the following value is found for this path length difference: 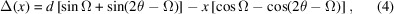
and we agree with this expression. A full calculation of the amplitudes based on this value would have given the conventional result, thus equivalent to the amplitude derived in reciprocal-space coordinates in the Appendix. The mistake that F14 makes is to demand (‘and must also be satisfied’) that this path length difference should be equal to
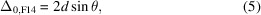
stated to be ‘the condition when 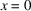
’. Demanding that these path length differences are the same means that
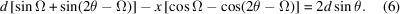
This is *only* true for 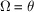. The true value for 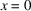 follows directly from equation (4): 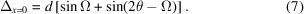
The values for 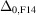 and 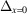 are again *only* equal when 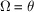. We fail to understand why this specific condition is needed, nor why one would expect Δ, which is found to be a function of *x*, to ever have the same value as at the point 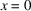. As already stated, the only exception for this is when 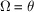, but the exercise is explicitly meant for conditions 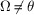. This is simply wrong. FW18 already notes this as the ‘first of the major errors’ in F14, but viewed this as an unjustified mathematical approximation. We consider this to be the conceptual error. Requiring Δ to be equal to 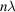 would make sense, because then the two points are in phase as was stated to be the aim of the computation, but that is not what F14 uses.

It is unclear how the erroneous path length difference leads to equation (F14-5), because no details are presented; but it is clear that the path length error explains the error in the final calculated amplitude, as discussed above. In the next section we will show how the path length difference can be calculated correctly, including the condition that parallel planes are in phase. We also show what the consequences are of the mistakes of F14.

### 2.3. No local maxima at 2θ_B_

Using the wrong expression for the amplitude, F14 comes to the conclusion that there is a peak in intensity at the Bragg scattering angle 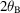, even when 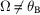, thus even when not at the Bragg condition. According to F14, this occurs because, at this condition, the parallel planes scatter in phase. We quote: ‘It is important to show that the scattering from a stack of parallel planes remains in phase when 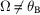 at the scattering angle 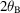
’. We will show that this is wrong and consider this to be the main misconception on which the new theory for X-ray diffraction was based.

To keep the discussion and mathematics simple, we will use the description using diffraction indices [equation (1)]. As discussed above, in F14 the phase difference between parallel planes is computed and a lateral distance *x* is introduced for which a point on the top plane is in phase with the underlying one. The argument in F14 is that when this is true for those two points, it will be true for all pairs of points and thus the planes are in phase. It is true that the planes can be in phase, but this does *not* occur at the Bragg scattering angle 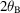 (except for the Bragg peak itself, when 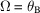). The condition for which the planes are in phase can be directly determined by using equation (1). The geometry we are considering corresponds to the 01 reflection in this 2D case. When we keep *k* at the value 1, then there is indeed no phase difference between parallel planes and the amplitude is 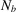 times the contribution of a single plane: 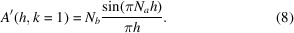
Equation (1) shows that the profile has a sharp maximum for 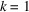, for any value of *h*. This local maximum thus occurs as a *straight* line in reciprocal space along the *h* direction. The value of the local maximum rapidly decreases away from the Bragg peak. (In Section 3 we will show that the local maximum is a feature specific for the parallelepiped crystal shape considered here.)

**Figure 3:**
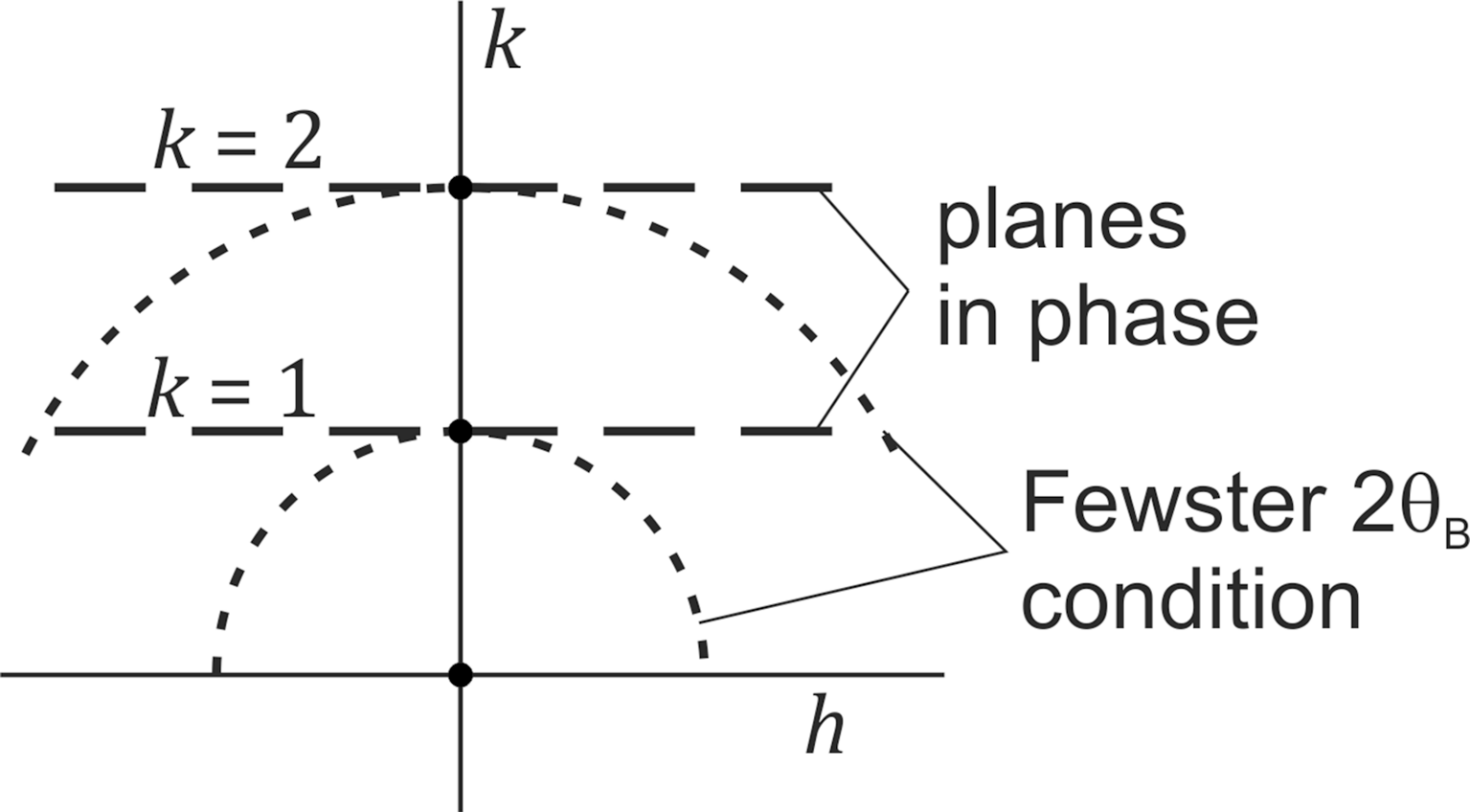
A schematic of reciprocal space showing the conditions for which the contributions of the parallel planes are in phase. This occurs for integer values of the diffraction index *k*. In F14, the in-phase condition is erroneously computed to lie along a circle.

The mistake in the path length difference in F14 leads to the erroneous result that the condition of perfect constructive interference between the planes corresponds to the condition that the scattering angle should be 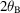. Using diffraction indices, this angular condition of F14 means that the length of the scattering vector should be 1. (The argument remains the same if we choose a different integer value for this diffraction index.) We can satisfy this by requiring 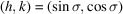, with σ an angle that represents the deviation from the Bragg condition. The trajectory of 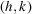 is thus a *circle* in reciprocal space and not the straight line at 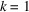 we found above. In short, F14 assumes that local maxima occur when 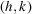 follows a circular trajectory, while in reality this occurs along a straight line for which 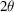 varies. Fig. 3 ▸ graphically shows the difference in these in-phase conditions. Very close to a Bragg peak, a circle is an excellent approximation of the straight line, but this circle is *not* the correct condition for constructive interference between the parallel planes. We note that Fig. 13 of FW18 illustrates the same issue, be it in angular coordinates.

Along this circle, the amplitude is
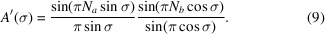
The right-most term shows that, for increasing values of σ (moving away from 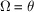), the planes are increasingly out of phase. This has two important consequences: (i) there is *no* local amplitude maximum at the scattering angle 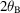, because this maximum moves to larger 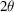 values when moving away from the Bragg peak, and (ii) there is *no* accumulation of significant intensity at the scattering angle 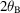 when a crystal is not oriented at the Bragg condition.

By considering a 2D case, with the amplitude as given in equation (1), F14 ignores another aspect of the intensity in reciprocal space. There is a local maximum along a straight *line* for 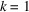 in this model. As will be discussed in more detail in the next section, for a 3D, cube-shaped crystal, a local maximum occurs any time two of the diffraction indices are integer, and thus the local maxima continue to occur along a *line*, also for the 3D case. In the 2D case a small fraction of grains of a powder sample may have the correct orientation to correspond to a local maximum, but in the 3D case with the additional degree of freedom, the fraction is much lower. The 2D case discussed in F14 thus creates the wrong impression of the actual situation in 3D.

We conclude that the ideas put forward in F14 are based on an error in the path length calculation and that the computation method corresponds to standard scattering theory. It is not a new theory for X-ray diffraction, but the conventional one, wrongly applied.

## 3. Crystal termination

We have found that in general there is no local amplitude maximum for the scattering angle equal to 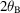, but there is certainly some intensity all over reciprocal space, away from the Bragg condition. This is predicted by conventional diffraction theory and equation (1) gives the amplitude for the 2D case when using planes instead of lattice points. In order to discuss the effect of the additional intensity, we will use the more realistic case of a 3D lattice. The derivation is done in Section *A*1 of the Appendix, with as result: 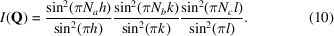
Here we left out pre-factors that are not important for this discussion. Note that we do not use the amplitude here, but the intensity. We will now discuss the significance of this intensity for a powder diffraction pattern. For simplicity, we will assume we have a crystal with a cubic shape and lattice.

**Figure 4:**
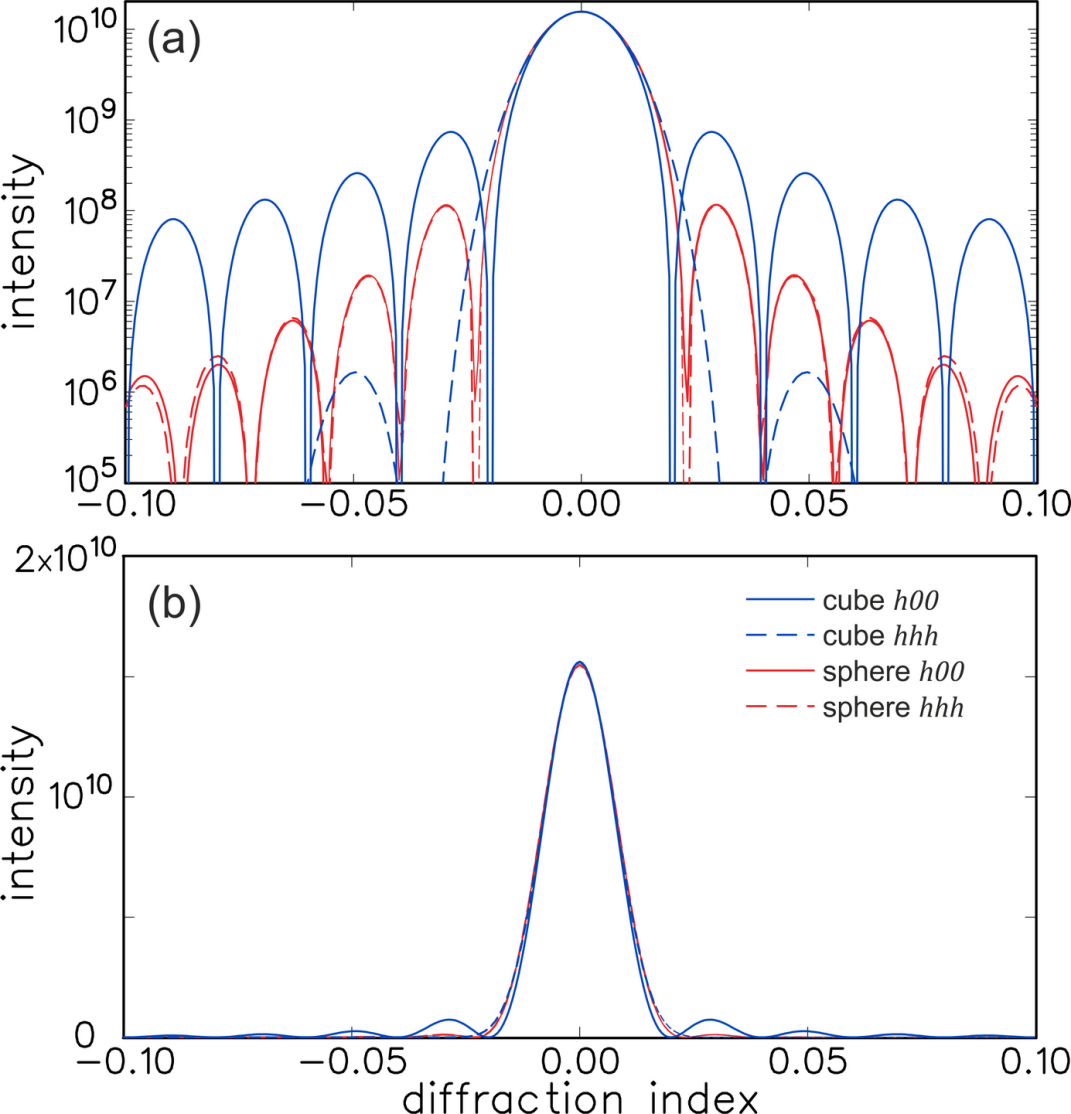
The intensity from a cubic crystal with a size of 50 unit cells (blue curves) and a spherical crystal with a radius of 31 unit cells (red curves). (*a*) Plotted on a log scale and (*b*) plotted on a linear scale. The graphs represent the intensity along the *h*00 and the *hhh* directions in reciprocal space. For the cubic crystal, these two intensities are very different, but for the spherical one they are nearly the same. On the log scale the secondary maxima appear significant, but the linear scale shows that these are very small.

Equation (10) gives very strong peaks when *hkl* are all integer, thus at the Bragg reflections. The equation also yields secondary maxima, where the strongest ones are found when two of the diffraction indices have integer values (meaning that along those directions the scattered waves are all in phase). This leads to spikes of intensity along the reciprocal-lattice axes, representing *local* maxima. For this specific crystal, these local maxima thus occur along six directions away from a Bragg peak. Along the *a* direction, for example, the first secondary maximum is at a distance 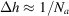 from the maximum and has a height that is a factor 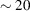 lower than the true maximum. Fig. 4 ▸ shows a plot along this direction for the case of 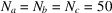 and 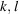 both integer, thus along the *h*00 direction in reciprocal space (solid blue curve). (For the cubic crystal considered here, the *h*00 direction in reciprocal space is the [100] direction in real space; in general the spikes will be perpendicular to the crystal facets.) For normal crystals, there are many unit cells and thus this secondary maximum is located very close to the main peak. Only in special circumstances can one observe these fringes, because almost always the angular and wavelength spread of the X-ray source will wash them out. (For thin films, where the number of layers can be very small, such fringes can be observed.) More importantly, however, is the fact that the intensity according to equation (10) is for the special case of a crystal with a very sharp termination along all six facets, leading to relatively strong maxima perpendicular to the facets. To illustrate the effect of these facets, the figure also shows the intensity along the *hhh* direction (dashed blue curve), thus not corresponding to a facet of this cubic shape. As expected, along this direction, the intensity is much weaker.

**Figure 5:**
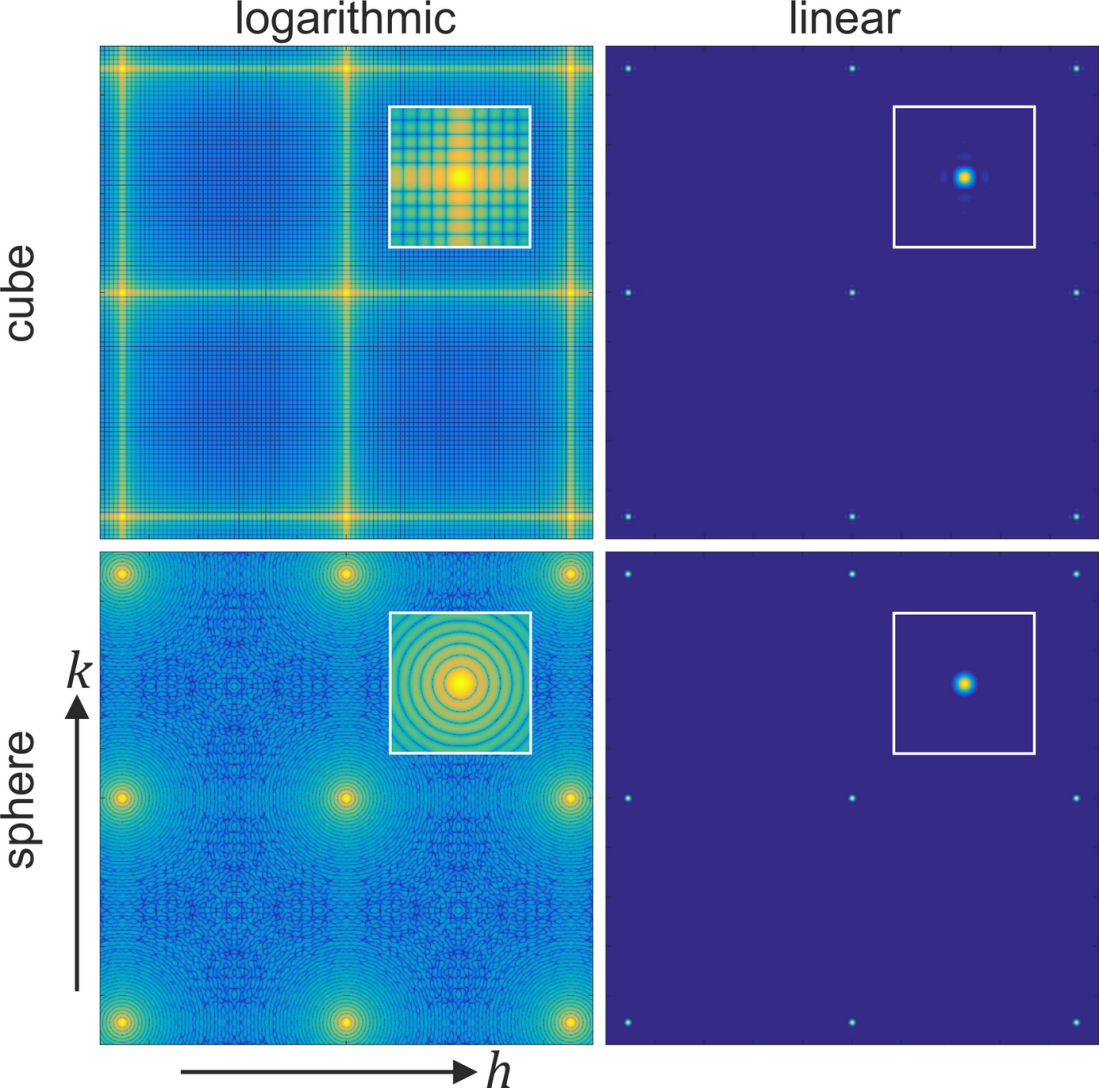
The intensity of crystals with cubic (top) and spherical (bottom) shape, plotted in the 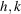 plane, and with *l* having an integer value. The sizes of the crystals are the same as in Fig. 4 ▸. For the cubic crystal, the intensity shows local maxima connecting the Bragg peaks, but this disappears for the spherical crystal. The plots using a logarithmic scale (left) clearly show the intensity distribution, but the linear scale (right) gives a more realistic idea of the residual intensity compared with the main peaks. The insets are an enlarged view of a single Bragg reflection.

An alternative illustration of these effects is shown in the top half of Fig. 5 ▸, where the intensity is plotted along a plane in reciprocal space. (A similar plot was presented in FW18.) The increased intensity when two of the diffraction indices are integer is clearly visible using a log scale. When plotted on a linear scale, however, the residual intensity is found to be very weak, even for this very small crystal. This was already visible more quantitatively in the line plots of Fig. 4 ▸.

The effect of the presence of well defined facets can be further illustrated by considering an alternative simple crystal form: a sphere. A sphere with a cubic lattice and with a radius of 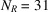 unit cells has the same volume as the 50 unit cell cube, so we use this size for comparison. The amplitude is now found from the summation
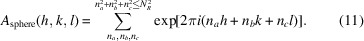
Because of the coupling of the three directions, this does not yield a simple intensity formula, but the summation can easily be done numerically. Fig. 4 ▸ plots the corresponding intensity for the *h*00 and *hhh* directions in reciprocal space (red curves). There are two main differences with respect to the cube: (i) the secondary maximum is much lower than for the cubic *h*00 direction (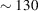 times weaker than the value of the main peak) and (ii) the *h*00 and *hhh* directions are now (nearly) equal. Both features arise from the fact that a sphere has no (large) facets and thus the secondary maxima are (nearly) equal in all directions and not peaked in any particular direction. While the secondary maxima are clearly visible in the log-scale plot, the linear scale shows that the secondary maxima are negligible compared with the real maximum and can thus be ignored. These conclusions are confirmed by the 2D intensity plot for the spherical crystal in Fig. 5 ▸. We note that also for the spherical crystal, the scattering amplitudes from different planes are in phase for integer values of the corresponding diffraction indices, but because of the shape, these directions do not lead to local maxima in reciprocal space. FW18 already discussed the case of a spherical crystal, using a description in angular coordinates.

Both a perfect cube and a perfect sphere are of course unrealistic representations of ‘normal’ crystals. When the shape is irregular, the secondary maxima are even weaker compared with the primary maximum. Besides the fact that the grains of a powder sample will typically have an irregular shape, they also have dimensions of some 10000 unit cells in all directions. This strongly reduces the residual intensity compared with the main peak, especially for crystals without large facets. We refer to Section *A*3 of the Appendix for details.

The secondary maxima will in general be further suppressed by the fact that typical surfaces will be rough. This is already somewhat illustrated in Fig. 4 ▸, because a sphere can (‘roughly’) be considered as a very rough version of a cube. A good and general way to demonstrate the effect of roughness is to use the so-called crystal shape function. The effect of surface roughness is a well known issue in the field of surface X-ray diffraction (Robinson & Tweet, 1992 ▸; Vlieg, 2012 ▸), where roughness can hamper the measurement of so-called crystal truncation rods (Robinson & Tweet, 1992 ▸). These rods are the equivalent of the intensity spikes of the cubic crystal, but without the fringes. The fringes are a finite-size effect, but crystal truncation rods occur also for half-infinite crystals, showing that the residual intensity can more generally be considered a crystal termination effect. Roughness will reduce the intensity near the Bragg peak by at least another factor 10. We refer to Section *A*4 of the Appendix for more details.

All these arguments show that the residual intensity, away from the Bragg condition, from a grain will be very low, of the order of 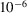 compared with the main peak. Having a powder with 1 million grains then might appear to still give significant intensity that will be added to the measured integrated intensity. However, this is not the case, because the crystals have random orientations and the intensity is distributed uniformly (for an ideal powder) over a sphere in reciprocal space. Only a small fraction of this will be in the part of the Debye–Scherrer ring that is observed. The integration of the intensity over all these grains at one specific observation direction is equivalent to the integration over the full reciprocal space of one grain. As we saw, only very close to the Bragg peak is there some intensity, but it is very weak. This means that the extra intensity remains at the level of 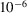. In F14, one of the motivations to explore the new theory was the observation of (quite) complete powder diffraction patterns from samples with about 300 grains. We have shown that the termination effects cannot explain this, but will come back to this interesting point in a future publication.

On individual grains, with the right size and shape and using a wide dynamic range, the tails (and even fringes) can be observed, and F18 shows some examples. This does not mean, however, that this intensity is significant for powder X-ray diffraction. We note that in F18, Fewster states (in his abstract) that the extra intensity is 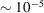. Fewster puts the intensity in the wrong place in reciprocal space, but the value mentioned in F18 agrees roughly with our estimates. In F14, however, the total contribution of the non-Bragg intensity at 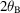 was estimated as 30%, but that is clearly in disagreement with the 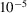 estimate Fewster made later, and also with our estimate.

Based on the wrong idea that conventional theory does not consider scattering from all crystal planes, F18 claims that FW18 have misunderstood the concept and methodology used in F14. F18 states that the new theory ‘only appears wrong to Fraser & Wark because the effect I predict has nothing to do with the crystal shape’. We want to stress here the point we have demonstrated above: when including the scattering from all planes, the resulting amplitude *does* depend on the crystal termination and thus on the crystal shape, because the planes have to end somewhere.

## 4. Conclusions

The ‘new theory for X-ray diffraction’ proposed by Fewster in 2014 is not new, but uses the standard methodology of X-ray scattering. Because of a conceptual error in the calculation of the path length difference, the application of the theory led to the wrong conclusion that there is a local maximum in scattered intensity at the Bragg scattering angle 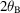, even from a crystal oriented away from the Bragg condition.

There is residual intensity in reciprocal space, away from the Bragg peaks, because of the termination of a crystal. The location and magnitude of this intensity depend on the size and shape of a crystal. Except for very special circumstances, the residual scattering does not give a significant intensity enhancement at the Bragg peaks in the case of powder X-ray diffraction. We thus have to discard the new theory and can continue to use the conventional one.

## Figures and Tables

**Figure 6:**
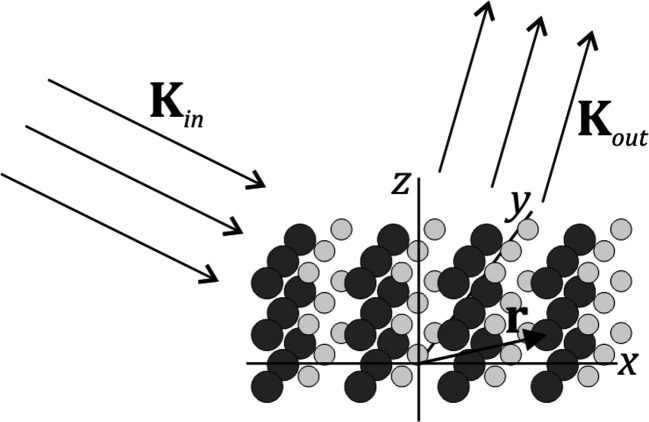
A schematic of the geometry when X-rays scatter from a crystal. The incoming and outgoing X-ray beams are assumed to be plane waves, represented by the wavevectors 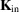 and 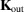, respectively. The scattering amplitude is obtained by summing over the contributions from all atoms.

**Figure 7:**
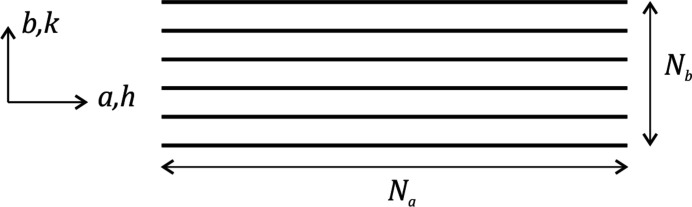
The 2D geometry considered in F14, using continuous planes (or lines in the 2D case) to represent the crystal lattice in the lateral direction.

**Figure 8:**
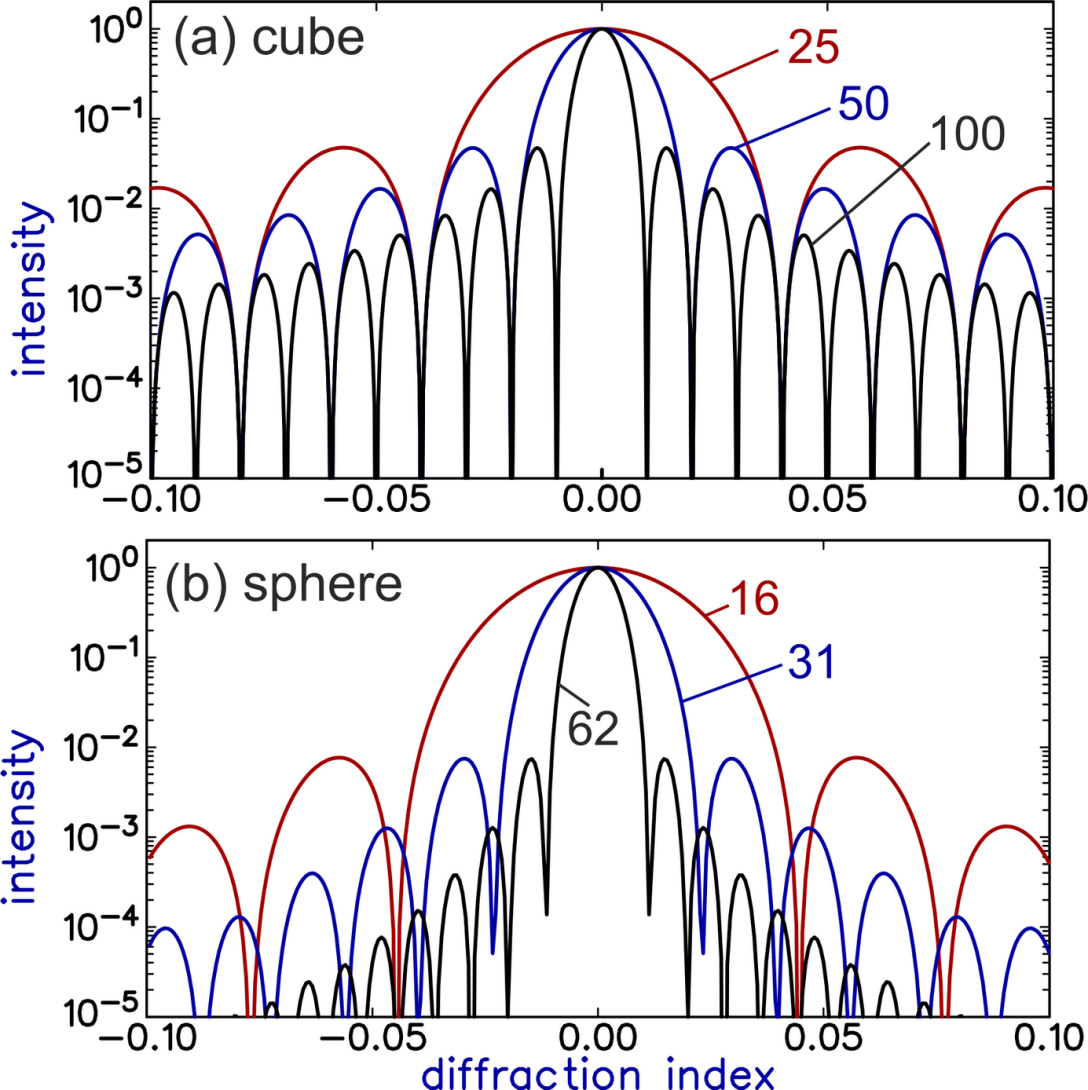
The normalized intensity profile of crystals with a cubic and with a spherical shape, for three different sizes: cubes with 25, 50 and 100 unit cells, and spheres with a radius of 16, 31 and 62 unit cells, chosen to have nearly the same volume as the cubes. The larger the crystal, the more rapid the decay of intensity away from the Bragg peak. In all cases the intensity is shown along the *h*00 direction in reciprocal space.

**Figure 9:**
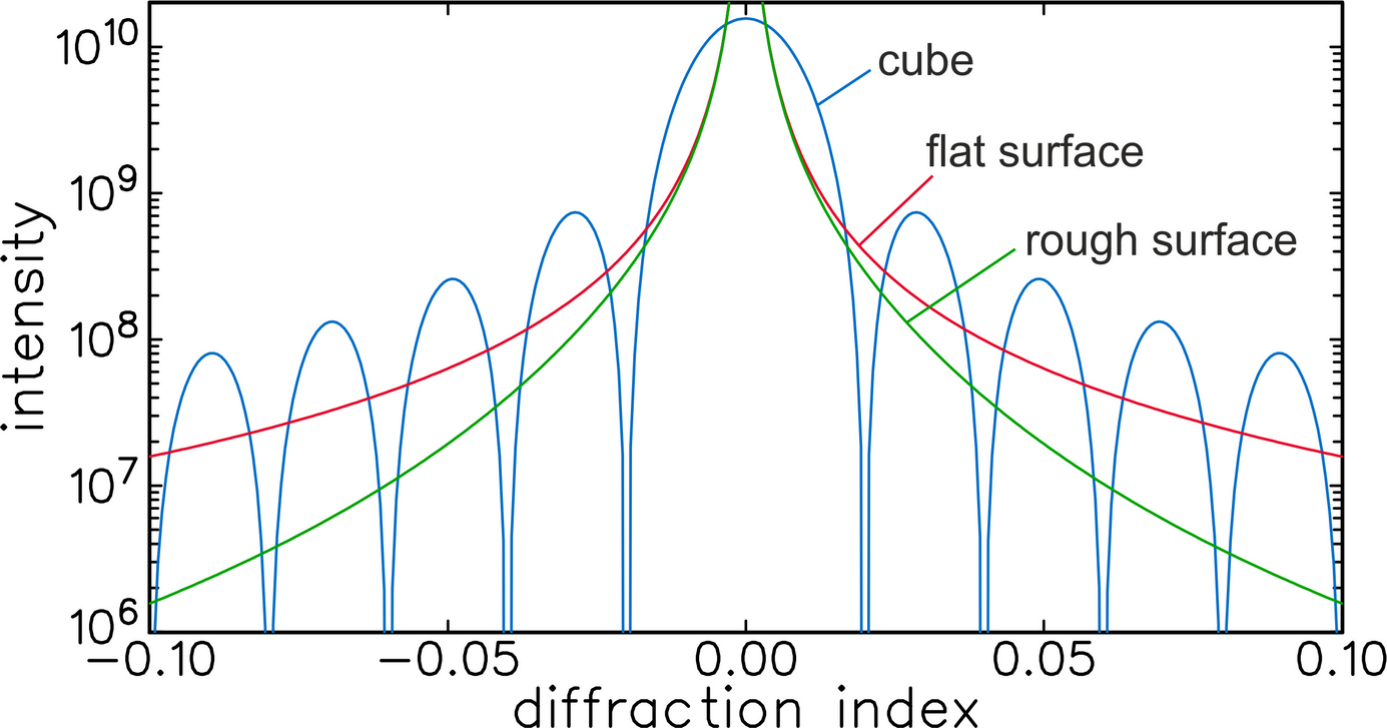
A comparison of the intensity from a cube-shaped crystal with that computed for a flat and a rough surface. The smooth curves are called crystal truncation rods in the context of surface diffraction. Roughness leads to a decrease in intensity.

## Appendix A

### A1. Kinematic scattering

We describe in some detail how the diffracted intensity from a crystal can be calculated. We present this for convenience to have all results at hand in a concise way, but do not claim any originality; the concepts discussed in this section can be found in standard textbooks. We use the perspective of X-ray scattering theory, which is somewhat different from the typical approach used by crystallographers, but the final results are the same. For the X-ray scattering approach we refer to the excellent treatments by Guinier (1994 ▸) and Cowley (1984 ▸). The crystallography approach can be found for example in the books by Warren (1990 ▸), Giacovazzo (2011 ▸) and Hammond (2015 ▸).

For small crystals, the scattering of X-rays is accurately described using the single-scattering approximation, also known as kinematic scattering. This means that the X-rays that are scattered from atoms are assumed not to scatter again. Since the scattering cross section of X-rays is very small, this is in most cases an excellent approximation. We further assume that we have a ‘perfect’ crystal, *i.e.* we ignore defects, because this is irrelevant for the issues we aim to address. When assuming, in addition, that the X-ray source and X-ray detector are at a large distance from the crystal, the incoming and outgoing beams can be described by plane waves. Then the so-called scattering amplitude can be written as

with *r_e_* the classical electron radius,  the momentum transfer,  the electron density and  a position within the crystal (with respect to a fixed origin). Fig. 6 ▸ schematically shows the scattering geometry. The length of the wavevectors is , with λ the wavelength. The scattering amplitude is the summation (integration) of the contributions of all atoms with the appropriate phase factor, given by . Mathematically, the amplitude is the Fourier transform of the electron density. The incoming () and outgoing () wavevectors are plane waves. Note that no assumption is made (yet) concerning the angles of the incoming or final wavevectors with respect to the crystal.

For a crystal, the atoms are located at well defined positions, as given by (i) the arrangement of the atoms within a unit cell and (ii) the periodic stacking of the unit cells according to the crystal lattice parameters *a*, *b* and *c*. In general, we can write the position of unit cell *j* as

with  and  integer values. The integral in equation (12) can thus be replaced by a summation over all unit cells and an integration over the electron density  of a single unit cell:

with

The summation in equation (14) can conveniently be performed by writing  as a vector in reciprocal space:

and where we explicitly allow the diffraction indices *h*, *k* and *l* to be real numbers, thus not restricted to integer values.  and  are the reciprocal-lattice vectors. Using this, the summation becomes

The result of this summation depends of course on the size and shape of the crystal. For simplicity, we start with a crystal in the form of a parallelepiped with  and  unit cells along the three crystal axes, respectively. The summation then becomes

These summations can be explicitly performed and written in a compact form. For the summation along the *a* axis, for example, we find

This shows that

and thus

The other summations can be done analogously, and therefore we obtain for the scattering amplitude

where we grouped the phase factors in :

This phase factor depends on the choice of origin, but disappears when we compute the intensity by multiplying the amplitude by its complex conjugate. Including the integral over the unit cell, we find for the intensity

This function has *very* sharp maxima when all diffraction indices have integer values. These maxima have values proportional to . The widths of the peaks are proportional to  along the three crystallographic directions, leading to an integrated intensity that is proportional to , *i.e.* proportional to the volume of the crystal. The fact that significant intensity is only found when *all* diffraction indices are integer means that the momentum transfer, equation (16), is a reciprocal-lattice vector :

where *H*, *K* and *L* are integer. (For clarity, we will thus use lower-case diffraction indices when these are meant to have real values, and upper-case when they are integer.) This also implies that the angles of the wavevectors with respect to the specific lattice plane (*HKL*) follow Bragg’s law. Bragg’s law is thus a consequence of the fact that strong intensity only occurs when the contributions of all unit cells are in phase and is not a pre-condition of the calculation. These consequences are shown here for the example of a crystal with parallelepiped shape, but remain valid for any crystal shape.

X-ray diffraction is frequently explained by considering the reflection of X-rays from a series of equally spaced planes with uniform intensity. This is a convenient way to derive Bragg’s law, but does not properly describe the physics of the scattering process. First of all, X-rays are *not* reflected from planes, but induce scattering from all atoms in the crystal and in all directions. The scattering from individual atoms is very weak, and thus only significant intensity occurs when the contributions of all unit cells are in phase. For a reflection *HKL* the condition that all contributions should be in phase is equivalent to the condition that the incoming and outgoing angles of the X-ray beams with respect to the (*HKL*) plane are equal, and thus this corresponds to a ‘reflection’. Secondly, the scattering centres are not distributed uniformly in a plane, but are discrete atoms arranged in a periodic lattice. For a correct calculation of the phase of each contribution, it is important to take this discrete nature into account, as was done in the derivation above.

We can, nevertheless, easily derive the scattering amplitude in the case of planes with uniform electron density. We assume the plane to be along the *a* and *b* directions. The summation over lattice points we performed above is replaced by an integral. For the *a* direction, this becomes

where  is the length of the plane in the *a* direction in fractional units. The amplitude of the full system is thus

Compared with the amplitude when the 3D lattice is included, the denominators for the *h* and *k* terms no longer have the sine function. With the continuous planes, Bragg peaks only occur at ; the others are absent, because no periodicity is included along the in-plane directions. It is somewhat un­natural to use a description in which there is a difference between the three crystallographic axes, because diffraction behaves the same in all directions. Nevertheless, the result when using the uniform plane approach is a good approximation when considering the scattering in the specular direction and its immediate vicinity.

In the main part of the paper, we compare the results of this conventional approach with those presented in F14. Since F14 uses the uniform plane approximation, we will do this as well. F14 also uses a 2D system, and it is straightforward to adapt our 3D result to this. By leaving out the *c* direction (*l* in reciprocal space), this result is valid for the 2D case when we swap *b* and *c*. The situation is sketched in Fig. 7 ▸. In order to be comparable with F14, we leave out the unimportant phase factors and also ignore the pre-factors in the amplitude, as these are not important for this comparison. The result of conventional diffraction theory for the 2D case is then

### A2. Conversion to angles

The amplitude in equation (28) is expressed in terms of (real) diffraction indices, which is a compact way of describing a position in reciprocal space. Each point  corresponds to a specific scattering geometry. F14 uses incoming and outgoing angles with respect to the scattering planes and therefore we will now convert equation (28) to angular coordinates.

Following F14, we use for the incoming angle the symbol Ω. The total scattering angle is fixed at , which may have a value corresponding to a Bragg reflection or not. The geometry is shown in Fig. 1 ▸. We can write the incoming and outgoing wavevectors as the following 2D vectors:

and

For the momentum transfer we then find

The last term is  written as a vector in reciprocal-space coordinates, and this yields

### A3. Crystal size

Fig. 4 ▸ showed the effect of the crystal shape on the residual intensity in reciprocal space. The relative intensity of the residual maxima strongly depends on the size of the crystals as well. Fig. 8 ▸ therefore shows the intensity for cubic and spherical crystals with different sizes (including the size used in the main text), normalized to a value of 1 at the Bragg peak. The figure shows that the relative intensity of the first secondary maximum is in all cases the same, but because the tails are much steeper, the intensity at a specific distance from the Bragg peak is rapidly decreasing for increasing crystal size. This is true for both crystal shapes, but for the spherical crystal the decrease as a function of size is much more pronounced. The decrease scales with the ratio of area over volume, where the area is that part of the crystal surface that has the orientation along the corresponding direction in reciprocal space. For the cubic shaped crystal, the facets are large and thus the *h*00 direction shows the weakest decay in intensity. Non-facet directions show a much stronger decay, similar to the spherical crystal shape.

The sizes shown in Fig. 8 ▸ are much smaller than typical grains of a powder sample, which will have sizes of 10000 unit cells or more. The residual intensity is then less than  of the Bragg peak.

### A4. Crystal termination and roughness effects

The effect of crystal termination and shape was demonstrated in the main text by considering the two special cases of a cubic or a spherical shape. These effects can be more generically shown by using the shape function  to describe the electron density of the crystal:

 was given in equation (13). Using this approach, the total electron density is written as the convolution of the electron density of a single unit cell  with the crystal lattice which is written as an infinite series of Dirac delta functions. This is multiplied by the shape function, which has value 1 inside the crystal and 0 elsewhere. Inserting this in equation (12) gives

where the pre-factors have been left out. This expression shows that the scattered amplitude is confined to points  in reciprocal space, with a weight given by the structure factor  and with a profile that is determined by the convolution with the Fourier transform  of the shape function. The intensity at each reciprocal-lattice point will thus be proportional to . As we already concluded above, the momentum transfer, equation (16), is strictly a reciprocal-lattice vector and the angles of the wavevectors with respect to the specific lattice plane (*HKL*) follow Bragg’s law.

We first apply the shape function and its Fourier transform to a crystal with a cubic shape. For a crystal with size  and  along the three axes (all in fractional, dimensionless coordinates), the shape function can be written as

The Fourier transform along the *x* direction is then

The *y* and *z* directions give equivalent results. This yields an amplitude that in essence is the same as in equation (22); only the sine in the denominator is not there [it is retrieved when doing a full summation over all lattice points (Vlieg, 2012 ▸), but that is unimportant here].  is the same as  if we assume its value is an integer. Using the shape function, we thus reproduce the result, equation (24), of the lattice summation we performed earlier.

Using the relation between shape function and its Fourier transform, we can now directly understand the effect of surface roughness. The cubic shape we just discussed corresponds to a *step* function along six directions and thus gives a relatively broad Fourier transform. However, facets of a typical crystal are not perfectly flat, but have a roughness, corresponding to a *broader* profile in real space. This means that the Fourier transform will be *sharper*, thus the tails become rapidly weaker away from the Bragg reflection.

The spherical shape can in principle also be treated using the shape function. We then need to compute the Fourier transform of a sphere, which, because of its abrupt termination, yields weak, spherical fringes in reciprocal space. For an irregular crystal shape, without significant facets and with surface roughness, the peak profile in reciprocal space becomes very sharp and the intensity away from the Bragg peak becomes so weak that it cannot be distinguished from the background. The integrated intensity of such a Bragg peak is thus a well defined quantity.

As we saw, the exact profile of a reflection depends on several details, and crystal termination is one of them. The fact that a sharply truncated crystal leads to extra intensity away from the main Bragg peaks is applied in surface X-ray diffraction (Robinson & Tweet, 1992 ▸; Vlieg, 2012 ▸), where crystal truncation rods (Robinson, 1986 ▸) represent the weak intensity tails emanating from Bragg peaks with a direction perpendicular to the surface. We can also use the shape function for this case. In surface diffraction, however, only one interface is normally probed, the other is so far away that the X-rays cannot reach this due to absorption. This can be accounted for by adding a (dimensionless) attenuation length Λ to the shape function. Following the convention in surface diffraction, we choose the *z* direction in real space (and the *l* direction in reciprocal space) for  to compute the scattering amplitude:

This gives for the Fourier transform:

Each reflection thus obtains tails of intensity that decay as . The fact that now only one interface plays a role means that no interference fringes occur, but only tails of monotonically decaying amplitude. The effect of Λ is only significant for *l* values very close to a bulk reflection, and is therefore often ignored (as in the rightmost part of the equation). The intensity for this crystal truncation rod for the case of an area of  unit cells (*i.e.* the cubic crystal we considered earlier) is plotted together with the cubic case in Fig. 9 ▸. We see that the decay follows that of the cubic, flat crystal. The local maxima of the cubic crystal are a factor 4 higher, because of the constructive interference of the amplitude of two interfaces for that case, compared with the amplitude of a single interface of the crystal truncation rod. The fact a single interface gives residual intensity in reciprocal space in the form of a crystal truncation rod shows that it is better to consider the residual intensity as a crystal termination effect, rather than a finite-size effect. For small crystals, the effects of two interfaces may lead to fringes, but that is not typical for crystal grains.

Typical surfaces are not perfectly flat, thus do not have the step profile given in equation (37). A mathematically convenient way to introduce surface roughness is to have an exponentially decaying roughness, characterized by a decay length ζ (in fractional, dimensionless coordinates). The shape function then becomes

Straightforward mathematics then yields for the amplitude along the *l* direction

where we again ignored the effect of the attenuation length Λ. The intensity is thus

Fig. 9 ▸ shows an example of the reduced intensity along a crystal truncation rod for , a value chosen to show a significant effect. The intensity decrease becomes more pronounced for increasing distance from the Bragg reflection. In surface X-ray diffraction one aims to measure intensity along the full crystal truncations rods, and thus it is important that the surface is as smooth as possible. A root-mean-square roughness of a few Å already makes it impossible to measure full crystal truncation rods. Also for nearly flat surfaces the intensity is weak (about a factor 1 million less than for bulk reflections) and thus synchrotron radiation sources are used to measure such rods and derive the surface structure from the integrated intensity. Crystals for which a surface is not specially prepared have no significant intensity tails, and this will be the case for typical grains of a powder sample.

A final point on the crystal termination concerns dynamical diffraction (Batterman & Cole, 1964 ▸). When deriving the diffracted intensity from a perfect (and large) crystal, multiple scattering plays a dominant role close to the Bragg condition and thus needs to be taken into account. (The Bragg angle in dynamical scattering differs slightly from that according to Bragg’s law because of refraction effects.) The theory shows that total scattering occurs over a very narrow angular range, beyond which the intensity rapidly decreases. These intensity tails show the same  dependence that we found for crystal truncation rods. This is as expected, because in the dynamical theory a flat surface is explicitly taken into account. The effects of multiple scattering rapidly decrease away from the Bragg condition and then the intensity becomes the same as that computed using the kinematic theory.
